# Effect and safety of oral Chinese patent medicine for heart failure: A protocol for systematic review and network meta-analysis

**DOI:** 10.1097/MD.0000000000022754

**Published:** 2020-10-16

**Authors:** Hui Guan, Guohua Dai, Ning Wang, Wulin Gao, Lili Ren, Zhenhao Cai

**Affiliations:** a First College of Clinical Medicine, Shandong University of Traditional Chinese Medicine; b Department of Cardiology, Affiliated Hospital of Shandong University of Traditional Chinese Medicine, Jinan, Shandong Province, China.

**Keywords:** Chinese patent medicine, heart failure, network meta-analysis

## Abstract

**Background:**

Heart failure (HF) is the terminal stage of various common cardiovascular diseases with quite a frequent readmission and a high mortality rate, and brings heavy financial burdens to families and society. Oral Chinese patent medicine (CPM) has been widely applied in the treatment of HF in China because of its simplicity, cheapness, convenience, and high efficiency. However, due to the large number and broad clinical selectivity of oral CPMs, there is a lack of uniformity and clinical application standardization. To choose more effective and safe medicine among so many oral CPMs is particularly essential for further improving the therapeutic effect. In this study, the efficacy and safety of different oral CPMs will be compared by a network meta-analysis (NMA), and the best CPM will be selected for the treatment of HF.

**Methods:**

According to the search strategy, 4 English and 4 Chinese databases will be searched from the construction of the library to July 31, 2020. The NMA will include clinical randomized controlled trials (RCTs) of different oral CPMs in HF treatment. The methodological quality is assessed according to the bias risk assessment tool of Cochrane. The Bayesian NMA is performed by Aggregate Data Drug Information System (ADDIS), and the results are visualized using Stata 15.0 software. The GRADE approach is used to assess the quality of evidence and recommendation intensity.

**Results:**

The NMA will identify the best oral CPM in the complementary treatment of HF. A peer-reviewed journal will publish the results of the study.

**Conclusion:**

This study can provide reliable evidence for the efficacy and safety of oral CPMs in the treatment of HF, and help decision-makers and patients to select more effective and safer oral CPM.

**Protocol registration number:**

INPLASY202090053.

## 1 Introduction

Heart failure (HF) is an end-stage of various heart diseases with high readmission and mortality rates, which brings heavy financial burdens to families and society. Although significant improvement has been achieved in concept and approaches of HF treatment, the readmission rate is still as high as 25% to 30% after being discharged 30 to 90 days,^[1,2]^ and the mortality rate of severe patients reaches more than 70% within 5 years.^[3]^ The prevention, treatment, and management of HF is a global public health problem.^[4]^

Traditional Chinese medicine (TCM) is an effective medicine with a history of 2000 years and widely used in the clinic. It shows apparent advantages in treating HF due to its holistic treatment, multitarget effects, personalized treatment by syndrome differentiation, enhancing physical condition, and reducing side effects of drugs.^[5–7]^ As previous studies confirmed, combination with TCM based on standardized western medicine treatment can improve symptoms, and reduce readmission and mortality rates of HF patients.^[8]^ Compared with Chinese herbal decoctions and injections, oral CPMs show advantages in carrying, cooking, odor, and using. Therefore, it is more suitable to take for a long time and improve HF patients’ compliance. More than 20 kinds of oral CPMs are applied in the HF treatment; to choose suitable and safe oral PCM is vital for improving treatment effects.

At present, there are no direct comparative studies of several oral CPMs in the HF treatment, and it is unrealistic to conduct new clinical trials. With direct and indirect comparisons, a systematic review and network meta-analysis can choose the best treatment plan from numerous interventions.^[9,10]^ This study will collect the RCTs of oral CPMs for HF treatment and determine the best oral CPM by screening, analyzing, ranking, and further provide reliable evidence for the clinical application of oral CPMs.

## 2 Methods

### 2.1 Protocol registration and ethical approval

This NMA is guided by the preferred reporting items for systematic review and meta-analysis protocols (PRISMA-P) 2015.^[11]^ The protocol has been registered on the INPLASY website, and the registration number is INPLASY202090053 (URL https://inplasy.com/inplasy-2020-9-0053/). The ethical approval and patient informed consent are abandoned because this study is based on published or registered RCTs.

### 2.2 Eligibility criteria

Eligibility criteria under the guidance of the PICOS principle include the following.^[12]^

#### 2.2.1 Participants

Patients diagnosed with HF according to any of the diagnostic criteria are eligible to be included, such as the 2014 “Guidelines for the Diagnosis and Treatment of Heart Failure in China,”^[13]^ the 2014 “Expert Consensus on TCM Diagnosis and Treatment of Chronic Heart Failure.”^[14]^ Patients who have received coronary artery bypass surgery or cardiac resynchronization therapy, or accompanied by noncardiovascular events such as malignant tumors, mental illnesses, or severe liver and kidney dysfunction are excluded. No restrictions are imposed on nationality, age, gender, and race.

#### 2.2.2 Interventions and comparators

The treatment group is defined as treating with oral CPMs based on western medicine, and the control group is defined as treating with western therapy alone or placebo or blank. According to the guidelines and expert consensus, oral CPMs mainly include Qili Qiangxin Capsules, Shexiang Baoxin Pills, Shengmai Capsules, Shenfu Qiangxin Pills, Tongxinluo Capsules, Xinbao Pills, Xuefu Zhuyu Capsules, Danshen Dripping Pills, Xinkeshu Pills, Zhenyuan Capsules, Qishen Yiqi Dripping Pills, etc. Western medicine mainly includes Ace inhibitors/Angiotensin II receptor antagonists, β-receptor blockers, aldosterone receptor antagonists, diuretics, nitrates, antiplatelet drugs, anti-myocardial ischemia drugs, and statins, etc.

#### 2.2.3 Outcomes

The primary efficacy outcomes contain mortality, other cardiovascular events; the secondary efficacy outcomes contain New York Heart Association classification (NYHA classification), left ventricular ejection fraction (LVEF), quality of life (QOL) scores, brain natriuretic peptide (BNP)/N-terminal pro-brain natriuretic peptide (NT-proBNP), 6-minute walk test (6-MWT), the safety indicators contain adverse events such as itchy skin or rash, nausea, vomiting, dizziness, dry cough, etc.

#### 2.2.4 Study of type

All published or ongoing RCTs of oral PCMs for HF will be included. Research sources mainly include journal papers, conference papers, and graduation thesis. The self-control and review literature, case report, experience summary, and repeatedly published literature is excluded.

### 2.3 Data sources

#### 2.3.1 Electronic search

According to the search strategy, 4 English databases include PubMed/MEDLINE, Web of Science, EMBASE, Cochrane Library, and 4 Chinese databases include China National Knowledge Infrastructure (CNKI), Wanfang Data, Chinese Scientific Journal Database, Chinses Biomedical Literature Database (CBM), which will be searched. The search time is from the establishment of the database to July 31, 2020. We will also search for ongoing RCTs, such as trials registered and conducted on WHO International Clinical Trial Registration Platform. The languages are restricted to Chinese and English.

#### 2.3.2 Other sources of data

In addition to a comprehensive search of the databases, manual search, tracking of references, and retrieving through search engines are also carried out.

### 2.4 Search strategy

The search terms include HF, Chinese patent medicine, Chinese herbal medicine, randomized controlled trial, and their synonyms. The strategy based on the combination of free-text terms and Medical Subject Heading (MeSH) is used for searching, and each database adopts a different search strategy according to its characteristics. Two members will develop and implement the search strategy independently, and a third member will further amend it. The detailed search strategy of PubMed is summarized in Table 1.

**Table 1:**
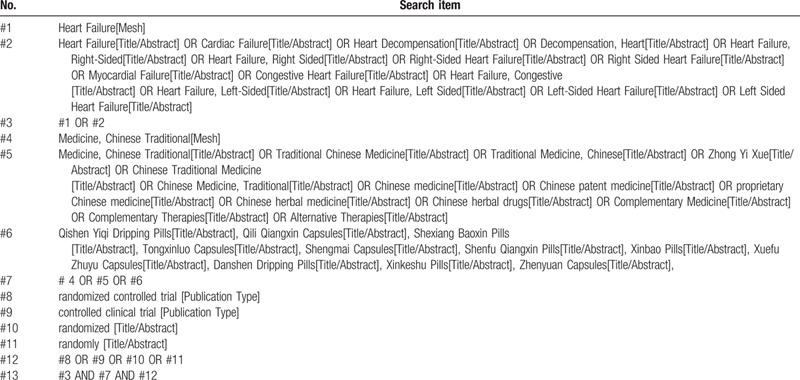
Search strategy for PubMed.

### 2.5 Literature screening and data extraction

The Endnote software (Endnote for Windows X9.0; Stanford, CT) is used to classify and organize and delete repeated literature. We will read the title and abstract to preliminarily screen and eliminate the literature that does not meet the requirements, and further read the full text to screen literature. Two members will perform literature screening independently, and if there are differences in opinion, they will discuss and resolve in consultation with the third member. The results of the literature screening will eventually be presented in the form of a flow chart. Two members are required to extract data independently with data extraction table, and then the data are input into statistical software after checking. The data extraction contents are as follows.

#### 2.5.1 Basic features of RCT

The basic features of RCT include title, first author, year of publication, country, journal.

#### 2.5.2 Participant characteristics

Participants characteristics include age, sex, inclusion/exclusion criteria, sample size, baseline characteristics.

#### 2.5.3 Interventions

Type of CPMs, treatment time, research design, randomization, distribution concealment and blindness.

#### 2.5.4 Outcomes

Primary efficacy outcomes, secondary efficacy outcomes, safety indicators.

#### 2.5.5 Bias risk assessment information

Study design and quality (using bias risk assessment tools).

### 2.6 Assessment of methodology quality

The methodological quality evaluation is one of the most important parts of the NMA, and it is the judgment of the authenticity of the research. The quality evaluation is mainly carried out by evaluating the bias risk in the design and implementation of clinical trials. All included literature is evaluated by the bias risk assessment tool of the Cochrane Handbook Version 5.2.0 for Systematic Reviews of Interventions, and 7 items are included in the tool. All biases are evaluated in 3 risk levels: low, uncertain, and high.^[15]^ For divergent literature evaluation results, independent evaluation and adjudication will be conducted by a third member.

### 2.7 Data synthesis and statistical methods

#### 2.7.1 Pairwise and network meta-analyses

In this study, the Stata software (Stata for Windows 15.0; Stata Corporation, College Station, TX) is used for pairwise meta-analysis. The odds ratio (OR) and mean difference (MD) is used as the effect sizes of continuous and categorical variables, respectively. The 95% confidence interval (95% CI) is calculated to represent the possibility of the results in this interval. The Aggregate Data Drug Information System (ADDIS) (Version for Windows 1.16.8) is used for NMA, and Markov-chain-Monte-Carlo (MCMC) is used to perform Bayesian inference. Perform iterative operations based on preset model parameters.^[16,17]^ After the statistical analysis, the convergence needs to be measured by the potential scale reduction factors (PSRE). The closer the PSRE is to 1, the better the convergence. PSRE greater than 1.1 or 1.2 means the number of simulations needs to be increased to achieve good convergence. After the consistency model analysis is completed, the rank probability and relative effects are used to compare the effects of CPMs.

#### 2.7.2 Assessment of heterogeneity

Before implementing the analysis, the clinical and methodological heterogeneity should be analyzed and identified. Only when the clinical and methodological features are similar enough can the effect size be synthesized. The heterogeneity is assessed for each pairing comparison using Stata 15.0 software. Chi-square test and *P* value are used to analyze statistical heterogeneity among the research results qualitatively, with *P* ≤ .05 indicating that there is statistical heterogeneity. *I*^2^ is used to analyze statistical heterogeneity quantitatively, with *I*^2^ > 50% indicating substantial heterogeneity. According to the heterogeneity assessment result, the fixed-effect model or random-effect model is selected to estimate the synthesized effect size.

#### 2.7.3 Sensitivity and subgroup analyses

In the process of effect size synthesizing, only when the included studies have the least heterogeneity, the credibility of the synthesized effect size is high. Sensitivity and subgroup analyses are the most common approaches to solve heterogeneity. Stata 15.0 software is used to analyze the sensitivity if the results of NMA are positive, and more than 3 studies are included. The sensitivity analysis is carried out by excluding study one by one. If no significant change exists in the results before and after the exclusion, it indicates that the sensitivity is low and the results are of stability and reliability; otherwise, it means a high sensitivity, and unstable results.^[18]^ It is necessary to analyze the causes if there is heterogeneity in the research results. Subgroup analysis will be carried out according to research characteristics related to the heterogeneity source. For example, if the heterogeneity may be caused by the methodology's quality, the hierarchical analysis may be carried out according to methodology quality; if different design schemes may cause it, the hierarchical analysis will be carried out according to the design scheme.

#### 2.7.4 Assessment of inconsistency

When there are closed loops in the NMA, the inconsistency is needed to assess. The Node-Split Model can calculate the differences between direct and indirect comparisons of evidence, and judge whether there is inconsistency according to the size of the *P* value.^[18]^ The *P* > .05 indicates that the results of the direct comparison are consistent with indirect comparison, and there is no statistical inconsistency.

#### 2.7.5 Publication bias

A funnel plot is used to assess the potential publication bias if the NMA includes 10 or more studies. The rank correlation test proposed by Begg or the linear regression method proposed by Egger is used to test the symmetry of the funnel plot. The X-axis of the funnel plot represents the effect size of each study, and the Y-axis represents the effect size's standard error. Taking the synthesized effect size as the central axis and making a vertical line on the X-axis. The point on the left side of the vertical line represents the effect size of the study is less than the synthesized effect size, and the point on the right represents the effect size is greater than the synthesized effect size. If the points on both sides are the same, it means the publication bias does not exist; otherwise, there is publication bias.^[16]^

### 2.8 Assessment of the quality of evidence

The GRADE approach is used for evaluating the quality and recommendation intensity of evidence.^[19]^ The quality of evidence refers to the degree to which people can determine the predicted value's correctness, and can be categorized into high, medium, low, and very low. Recommendation intensity refers to the extent to which you can be confident that compliance with recommendations outweighs disadvantages, and can be divided into 2 levels: strength and weakness. The basic application principle of GRADE approach in NMA mainly includes 5 downgrade factors.^[20]^

## 3 Discussion

With the continuous updating of evidence-based therapies in recent years, Ace inhibitors/Angiotensin II receptor antagonists, β-receptor blockers, and aldosterone receptor antagonists have become common drugs for HF, which can effectively improve clinical symptoms and patients’ life quality. However, the prognosis of HF patients is still unfavorable. Exploring effective treatment methods, improving HF patients’ prognosis is a public health issue of global concern.^[4,21]^ TCM is an effective supplement and substitute for primary and secondary prevention of cardiovascular diseases.^[22]^ It can improve HF patients’ cardiac function and life quality as adjuvant therapy. Among all kinds of TCM, oral PCMs have been widely applied in HF treatment due to its portability, convenience, low cost, and high efficiency in China. Previous systematic reviews and meta analyses on Qili Qiangxin capsules, Qishen Yiqi dripping pills, Shexiang Baoxin pills, Wenxin granules, and other oral PCMs show good efficacy and safety in the treatment of HF.^[16,23–25]^

Most previous studies focus on the clinical efficacy comparison of one oral PCM and western medicine in the treatment of HF. There is still a lack of a comparison of clinical efficacy and safety of several PCMs. There are many kinds of oral PCMs in the clinic, which shows a better effect in the treatment of HF is still unknown. In this study, an NMA is conducted by comparing primary efficacy outcomes, secondary efficacy outcomes, safety indicators of all RCTs of oral PCMs in the treatment of HF, and the probability is further ranked according to the indicators. This NMA aims to select the best clinical medicine for the treatment of HF with oral PCMs.

This study has several disadvantages: First, we have taken various measures to improve the searching quality as much as possible. However, due to the impossibility of searching all databases and insufficient search of unpublished studies, there may still be incomprehensive literature. Second, the published RCTs of oral PCMs in the treatment of HF lack strict randomized design, and with low quality, this may reduce the credibility of the study. Third, only English and Chinese literature is included in this study, which may lead to selection bias. Anyway, this study can provide a reliable result for the best medicine selection and strong evidence for the significant advantages of oral PCMs in the treatment of HF.

## Author contributions

**Conceptualization:** Hui Guan, Hua-hua Dai.

**Data curation:** Hui Guan, Wulin Gao, Ning Wang, Lili Ren, Zhenhao Cai

**Formal analysis:** Hui Guan, Ning Wang, Lili Ren, Zhenhao Cai.

**Funding acquisition:** Hua-hua Dai.

**Methodology:** Hui Guan, Wulin Gao.

**Project administration:** Hua-hua Dai.

**Writing – original draft:** Hui Guan, Ning Wang.

**Writing – review & editing:** Hui Guan, Hua-hua Dai, Guohua Dai.
